# Successful Transitions to Remote Assessments and Technology Installations due to COVID-19: The I-CONECT Project

**DOI:** 10.1093/geroni/igaa057.3502

**Published:** 2020-12-16

**Authors:** Khoa Nguyen, Mattie McDonald, Colton Scavone, Nora Mattek, Jeffrey Kaye, Hiroko Dodge

**Affiliations:** 1 Oregon Health & Science University, Portland, Oregon, United States; 2 Layton Alzheimer’s Disease Center, Portland, Oregon, United States; 3 Pregon Health & Science University, Portland, Oregon, United States

## Abstract

I-CONECT is a randomized controlled clinical trial to examine the impact of social interaction delivered via video-chat on cognitive function (clinicaltrials.gov number: NCT02871921, project website: www.I-CONECT.org ). We aimed to enroll 320 community-dwelling socially isolated older adults (age >=75 years). The recruitment of participants has started in 2018 and was ongoing when COVID-19 pandemic began. Video chat and telephone-based social interaction interventions did not change during COVID-19. However, new recruitment and cognitive assessments, which require in-person contact and deployment and retrieval of video chat devices in participant homes, were suspended due to the nature of our study population (i.e., older age, higher likelihood of comorbidities). Recently we were able to successfully switch to complete remote assessments including 1) telephone-based cognitive assessments using T-COG (Telephone Cognitive Assessment battery), and 2) contactless delivery of our study devices (Chrome books and electronic pill boxes) for subject self-installation. Our creative approach to self-installations includes color coded pictures and an easy-to-follow installation manual, accompanied by remote instruction and support via telephone. This poster introduces our remote assessment and installation protocol and participant and technical support team feedback regarding this new contactless protocol. This presentation provides useful guidance for future studies considering completely remote assessment and telemedicine approaches.

